# Effect of glucose variability on the mortality of adults aged 75 years and over during the first year of the COVID-19 pandemic

**DOI:** 10.1186/s12877-024-05149-0

**Published:** 2024-06-20

**Authors:** Miguel A. Salinero-Fort, F. Javier San Andrés-Rebollo, Juan Cárdenas-Valladolid, José Mostaza, Carlos Lahoz, Fernando Rodriguez-Artalejo, Paloma Gómez-Campelo, Pilar Vich-Pérez, Rodrigo Jiménez-García, José M. de-Miguel-Yanes, Javier Maroto-Rodriguez, Belén Taulero-Escalera, Víctor Iriarte Campo, on behalf of the Aged-Madrid Study Research Group, A. Gutiérrez-Misis, E. Carrillo de Santa Pau, M. V. Castell-Alcalá, B. Álvarez-Embarba, N. Behzadi-Koochani, C. de Burgos-Lunar, P. Regueiro-Toribio, T. Gijón-Conde

**Affiliations:** 1 Department of Health, Foundation for Biosanitary Research and Innovation in Primary Care, The Hospital La Paz Institute for Health Research (IdiPAZ), Alfonso X El Sabio University, Research Network On Chronicity, Primary Care and Health Promotion -RICAPPS-(RICORS), General Subdirectorate of Research and Documentation, Madrid, Spain; 2 Foundation for Biosanitary Research and Innovation in Primary Care, Las Calesas Health Center, Madrid, Spain; 3 Foundation for Biosanitary Research and Innovation in Primary Care, Information Systems Department, Primary Health Care Management of Madrid, Alfonso X El Sabio University, The Hospital La Paz Institute for Health Research (IdiPAZ), Madrid, Spain; 4 Lipids and Vascular Risk Unit, Internal Medicine, University Hospital La Paz-Cantoblanco-Carlos III, The Hospital La Paz Institute for Health Research (IdiPAZ), Madrid, Spain; 5 Department of Preventive Medicine and Public Health, Universidad Autónoma de Madrid-IdIPAZ, CIBERESP (CIBER of Epidemiology and Public Health), and IMDEA-Food Institute, CEI UAM+CSIC, Madrid, Spain; 6 Foundation for Biomedical Research of La Paz University Hospital (FIBHULP), The Hospital La Paz Institute for Health Research (IdiPAZ), Madrid, Spain; 7 Foundation for Biosanitary Research and Innovation in Primary Care, Los Alpes Health Center, Madrid, Spain; 8 Department of Public Health & Maternal and Child Health, Faculty of Medicine, Universidad Complutense de Madrid, Madrid, 28040 Spain; 9 School of Medicine, Internal Medicine Department, Complutense University of Madrid, Gregorio Marañón General University Hospital, Gregorio Marañón Health Research Institute (IiSGM), Madrid, Spain; 10 Department of Preventive Medicine and Public Health, School of Medicine, Universidad Autónoma de Madrid, Calle del Arzobispo Morcillo 4, Madrid, 28029 Spain; 11 Foundation for Biosanitary Research and Innovation in Primary Care, Madrid, Spain; 12 Subdirección General de Investigación Sanitaria, Consejería de Sanidad, Madrid, Spain

**Keywords:** Blood glucose, Age, Mortality, Follow-up studies, COVID-19

## Abstract

**Background:**

To our knowledge, only one study has examined the association between glucose variability (GV) and mortality in the elderly population with diabetes. GV was assessed by HbA1c, and a J-shaped curve was observed in the relationship between HbA1c thresholds and mortality. No study of GV was conducted during the COVID-19 pandemic and its lockdown. This study aims to evaluate whether GV is an independent predictor of all-cause mortality in patients aged 75 years or older with and without COVID-19 who were followed during the first year of the COVID-19 pandemic and its lockdown measures.

**Methods:**

This was a retrospective cohort study of 407,492 patients from the AGED-MADRID dataset aged 83.5 (SD 5.8) years; 63.2% were women, and 29.3% had diabetes. GV was measured by the coefficient of variation of fasting plasma glucose (CV-FPG) over 6 years of follow-up (2015–2020). The outcome measure was all-cause mortality in 2020. Four models of logistic regression were performed, from simple (age, sex) to fully adjusted, to assess the effect of CV-FPG on all-cause mortality.

**Results:**

During follow-up, 34,925 patients died (14,999 women and 19,926 men), with an all-cause mortality rate of 822.3 per 10,000 person-years (95% confidence interval (CI), 813.7 to 822.3) (739 per 10,000; 95% CI 728.7 to 739.0 in women and 967.1 per 10,000; 95% CI 951.7 to 967.2 in men). The highest quartile of CV-FPG was significantly more common in the deceased group (40.1% vs. 23.6%; *p* < 0.001). In the fully adjusted model including dementia (Alzheimer’s disease) and basal FPG, the odds ratio for mortality ranged from 1.88 to 2.06 in patients with T2DM and from 2.30 to 2.61 in patients with normoglycaemia, according to different sensitivity analyses.

**Conclusions:**

GV has clear implications for clinical practice, as its assessment as a risk prediction tool should be included in the routine follow-up of the elderly and in a comprehensive geriatric assessment. Electronic health records can incorporate tools that allow its calculation, and with this information, clinicians will have a broader view of the medium- and long-term prognosis of their patients**.**

**Supplementary Information:**

The online version contains supplementary material available at 10.1186/s12877-024-05149-0.

## Background

Several studies have analysed mortality in elderly individuals, with cardiovascular and oncological as the most frequent causes of death in the developed world [1]. The crude mortality rate for the elderly in Spain was 3,824 deaths per 100,000 inhabitants aged 75–84 (2017) [2]. Factors such as socioeconomic level [3], frailty [4], physical activity [5, 6], self-rated health status [7], social and family support [8], chronic diseases [9, 10], multimorbidity [11], nutritional status [12], body mass index [11], and cognitive function [13] may influence mortality rates. However, it is unknown whether any homeostatic factor that influences the degree of control of chronic diseases may be associated with an increased all-cause mortality in the elderly population.

Day-to-day glucose variability (GV) is considered a homeostatic phenomenon defined as the oscillation of blood glucose levels outside the normal range, which is a predictor of microvascular and macrovascular diseases and all-cause mortality, particularly in patients with type 2 diabetes mellitus (T2DM) [14, 15]. Our initial findings confirmed a clear association between GV, as measured by the coefficient of variation of fasting plasma glucose (FPG), and all-cause mortality in patients with T2DM and additionally in individuals with prediabetes or normoglycemia [16].

In 2020, the SARS-CoV-2 lockdown and social distancing motivated changes in the general population’s daily routines, specifically in elderly people, favouring an unbalanced diet, less physical activity, unavailability of some medications [17], and significantly less contact with health care professionals than usual [18]. We hypothesize that this situation could worsen the control of chronic diseases in elderly patients with a history of GV and favour an increase in all-cause mortality.

This study aims to evaluate whether GV, mediated by oxidative stress and other factors associated with senescence, is an independent predictor of all-cause mortality in patients aged 75 years or older with and without COVID-19, followed during the first year of the COVID-19 pandemic and its lockdown measures in Spain.

## Material and methods

A retrospective cohort study was carried out in the Aged-Madrid Study, a new data analytics platform in Madrid (Spain) created to address urgent COVID-19-related questions. We used routinely collected electronic health records (EHRs) from primary care practices using AP-Madrid software, covering 424 practices (3,881 general practitioners) and 100% of the population in Madrid, linked to the Office of National Statistics death registrations (INDEF). We included all adults (aged 75 years or over) alive and under follow-up on 1 January 2020 and with at least five years of continuous EHRs in primary care before this date. We ensured that baseline data could be adequately captured (*n* = 587,603). We excluded individuals with missing values for age, sex or less than three FPG values between 2015 and 2020. We directly compared both all-cause mortality and COVID-19 mortality with survivors to identify the covariates to include in the models. The variable of most interest was GV, which was used to test the main hypothesis.

The CV-FPG was obtained in those patients with at least three values of FPG, with an interval between FPG measurements of at least twelve months, along a follow-up of six years and calculated as the ratio of the standard deviation to the mean FPG multiplied by 100. Patients were categorized according to the quartiles of CV-FPG. The values of these quartiles were Q1: ≤ 5.2635; Q2: 5.2636 to 7.8577; Q3: 7.8578 to 12.3739; and Q4: ≥ 12.3740.

We considered SARS-CoV-2 death when it was registered as such in the clinical chart of a hospitalized patient or when the death recorded in the INDEF occurred 15 days from a first confirmed diagnosis of SARS-CoV-2 infection. The INDEF collects all deaths occurring in Spain, but it does not record their cause.

Covariates considered in the analysis included age, sex, cardiovascular risk factors, morbidities, and medication prescriptions until December 31, 2019, and were obtained from EHRs. We recorded morbidities according to the International Classification of Primary Care (ICPC-2). We specifically registered the presence of any previous cardiovascular disease (either myocardial infarction, angina, stroke, or peripheral artery disease), any cancer active during the previous five years (except nonmelanoma skin cancer), chronic kidney disease (CKD), congestive heart failure, chronic obstructive pulmonary disease (COPD), atrial fibrillation, dementia (Alzheimer’s disease), hypertension, and diabetes. We also gathered information about tobacco consumption from EHRs.

**Fig. 1:**
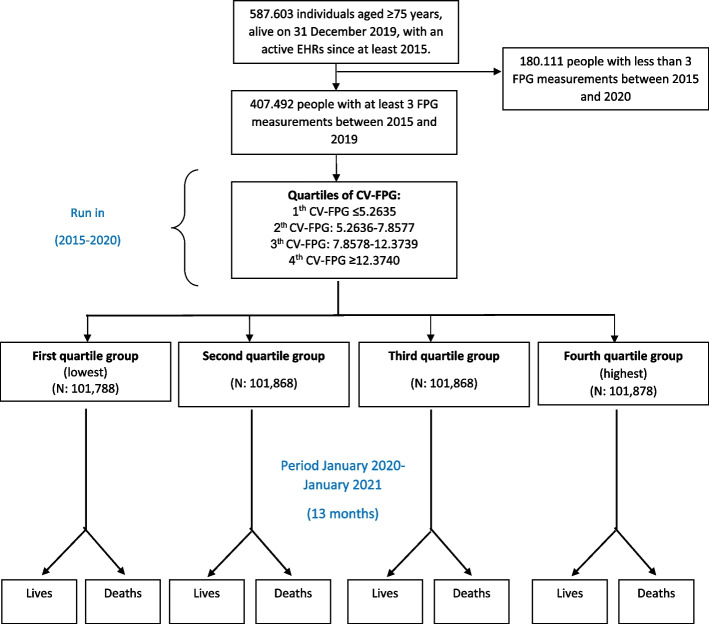
Study flow diagram

All blood analyses and anthropometric measurements performed between January 1, 2015 and December 31, 2020 were available for the study. However, FPG values and other biochemical parameters measured during hospitalization were not used to avoid artifacts in the results, as their values may depend on the reason for hospitalization or the cause of death at the time of hospitalization. A total of 180,111 patients were excluded for having < 3 FPG measurements during follow-up, and this analysis was performed on 407,492 patients (Fig. 1).

Medications prescribed for chronic medical conditions were obtained from the Electronic Pharmacy Database (Módulo Unico de Prescripción, MUP) of Madrid, integrated in EHRs.

There were no losses to mortality follow-up because regardless of whether the patient moved to a new city, the mortality registry is at the national level and is based on the patient’s identification data, including the national identity card number, which is unique for each Spanish citizen.

We have validated the quality of the EHRs in primary care for research use [19–21], and the database has been widely employed to study the epidemiology of cardiovascular risk factors in older patients [22].

### Statistical analysis

We used unpaired Student’s t test or one-way analysis of variance for continuous variables and chi-squared test for categorical variables for comparisons between/among subgroups. Mortality rates were calculated per 10,000 person-years with their 95% confidence interval (CI). These mortality rates were stratified by quartiles of CV-FPG and glycemic status.

Univariate and multivariate survival analyses were performed by logistic regression. In the first analysis for all-cause mortality, the odds ratios (ORs) and 95% CIs were calculated based on model 1: adjusted for age and sex; model 2: adjusted for age, sex, history of cardiovascular disease, heart failure, COVID-19 infection, and cancer; model 3: adjusted for variables in model 2 plus glycemic status, hypertension, atrial fibrillation, COPD, CKD, use of tobacco, dyslipidemia, use of antiplatelet drugs, statins, and dual blockade of the renin–angiotensin–aldosterone system (RAAS); and model 4: model 3 plus dementia (Alzheimer’s disease), mean FPG and stratified by glycemic status (normoglycemia, T1DM and T2DM) when the interaction between quartiles of CV-FPG and glycemic status was positive (*p* < 0.05).

Finally, three sensitivity analyses were performed. The first excluded participants with cancer in the previous two years to avoid its possible influence on mortality. The second we excluded patients who died of COVID-19. The third one included subjects with COVID-19 infection and without history of cancer.

Analyses were performed with SPSS version 21.0 (IBM Corp., Chicago, IL) and Epidat 4.1 software; a 2-sided *p* value < 0.05 was considered statistically significant.

## Results

Table 1 shows the baseline sociodemographic, anthropometric, and clinical factors of 407,492 participants aged 83.5 (SD 5.7) years; 63.2% were women, and 29.3% had diabetes. There were significant differences between women and men in age, Barthel Index, dementia (Alzheimer’s disease), smoking habits, cardiovascular risk factors (dyslipidemia, hypertension, diabetes mellitus), cardiovascular diseases (myocardial infarction, stroke, peripheral artery disease), and heart failure.

**Table 1:** Baseline characteristics of the population aged 75 and over (1st January 2020)

	**All (*N* = 407,492)**	**Women (*N* = 257,401)**	**Men (*N* = 150,091)**	***p* value**
Age (years), mean (SD)	83.5 (5.7)	83.9 (5.8)	82.7 (5.3)	< 0.001
**Age group**				
75–84 y, n (%)	244.946 (60.1)	146,377 (56.9)	98,569 (65.7)	< 0.001
85–94 y, n (%)	146.228 (35.9)	98,343 (38.2)	47,885 (31.9)	
> 94 y, n (%)	16,318 (4)	12,681 (4.9)	3,637 (2.8)	
BMI, mean (SD)	28.6 (4.7)	28.8 (5.1)	28.4 (4)	< 0.001
Barthel index, mean (SD)	78.8 (21.1)	78.1 (21.3)	80.4 (20.7)	< 0.001
**Barthel group**				
Barthel: Total dependence, n/N (%)	6,559/159,720 (4.1)	4,623/111,950 (4.1)	1,936/47,770 (4.1)	< 0.001
Barthel: severe dependence, n/N (%)	6,343/159,720 (4)	4,721/111,950 (4.2)	1,622/47,770 (3.4)	
Barthel: moderate dependence, n/N (%)	14,054/159,720 (8.8)	10,589/111,950 (9.5)	3,465/47,770 (7.3)	
Barthel: mild dependence, n/N (%)	132,760/159,720 (83.1)	92,015/111,950 (82.2)	40,745/47,770 (85.3)	
Current Smoking, n (%)	20,867 (5.1)	7,137 (2.8)	13,730 (9.1)	< 0.001
Baseline SBP, mean (SD)	132.7 (16.2)	133 (16.3)	132.3 (16.1)	< 0.001
Baseline DBP, mean (SD)	73.8 (9.5)	74 (9.4)	73.5 (9.5)	< 0.001
Dementia (Alzheimer’s disease), n (%)	34.100 (8.4)	24.501 (9.5)	9,599 (6.4)	< 0.001
Dyslipidemia, n (%)	244,365 (60)	162,711 (63.2)	81,654 (54.4)	< 0.001
Hypertension, n (%)	301,608 (74)	196,780 (76.4)	104,828 (69.8)	< 0.001
T1DM, n (%)	1,676 (0.4)	999 (0.4)	677 (0.5)	0.002
T2DM, n (%)	117.880 (28.9)	66,824 (26)	51,166 (34)	< 0.001
T2DM + Hypertension, n (%)	96,070 (23.6)	57,598 (22.4)	38,472 (25.6)	< 0.001
**CVD, n (%)**	60,573 (14.9)	26,637 (10.4)	33,936 (22.6)	< 0.001
1 vascular bed	54,952 (13.5)	25,157 (9.8)	29,795 (19.9)	< 0.001
2 vascular beds	5,353 (1.3)	1,433 (0.6)	3,920 (2.6)	
3 vascular beds	268 (0.1)	47 (0.0)	221 (0.1)	
Heart failure, n (%)	29,110 (7.1)	19,096 (7.4)	10,014 (6.7)	< 0.001
CKD, n (%)	124,529 (30.6)	76,887 (29.9)	47,642 (31.7)	< 0.001
Number of glucose measurements, mean (SD)	5.89 (2.7)	5.94 (2.7)	5.81 (2.7)	< 0.001
**FPG, mean (SD) **[when at least 3 FPG measurements]	103.3 (24.3)	101.4 (23.8)	106.4 (24.7)	< 0.001
T2DM patients	129 (26.9)	128.5 (27.7)	129.6 (25.9)	< 0.001
T1DM patients	137.4 (35.7)	138.5 (36.3)	135.8 (34.8)	0.114
Non-DM patients	92.5 (11.7)	91.7 (10.5)	94.2 (11.9)	< 0.001
**CV-FPG, mean (SD) **[when at least 3 FPG measurements]	10.5 (8.7)	10.2 (8.6)	10.8 (9)	< 0.001
T2DM patients	17.20 (11.53)	17.46 (11.69)	16.86 (11.31)	< 0.001
T1DM patients	27.55 (14.73)	28.47 (15.02)	26.20 (14.20)	0.002
Non-DM patients	7.61 (4.9)	7.60 (4.86)	7.61 (4.97)	0.696
**FPG < 60 mg/dl at least 20% of measurements (%)**^**a**^	2,790 (0.5)	1,592 (0.6)	794 (0.5)	< 0.001
T2DM patients, n/N (%)	1,316/117,880 (1.1)	826/66,824 (1.2)	490/51,056 (1)	< 0.001
T1DM patients, n/N (%)	78/1,676 (4.7)	51/999 (5.1)	27/677 (4)	0.345
Non-DM patients, n/N (%)	992/287,936 (0.3)	715/189,578 (0.4)	277/98,358 (0.3)	< 0.001
**HbA1c (%), mean (SD)**^**b**^** [when at least 3 HbA1c measurements]**	6.38 (0.9)	6.35 (0.9)	6.43 (0.9)	< 0.001
T2DM patients	6.88 (0.9)	6.89 (0.9)	6.86 (0.9)	< 0.001
T1DM patients	7.54 (1)	7.64 (1)	7.42 (1)	0.002
Non-DM patients	5.77 (0.4)	5.77 (0.4)	5.78 (0.4)	0.204

*CVD* cardiovascular disease (myocardial infarction, stroke, or peripheral artery disease), *CKD* chronic kidney disease (CKD-EPI < 60 ml/min/1.73 m^2^ and/or albumin/creatinine ratio ≥ 30 mg/g (≥ 3 mg/mmol)), *FPG* fasting plasma glucose, *CV-FPG* coefficient of variation of fasting plasma glucose

^a^If the relative frequency of each patient with FPG below 60 mg/dl was 20% or more during 2015–2020

^b^Mean HbA1c was calculated as the mean of all HbA1c measurements if at least three measurements were taken between 2015 and 2020

**Fig. 2:**
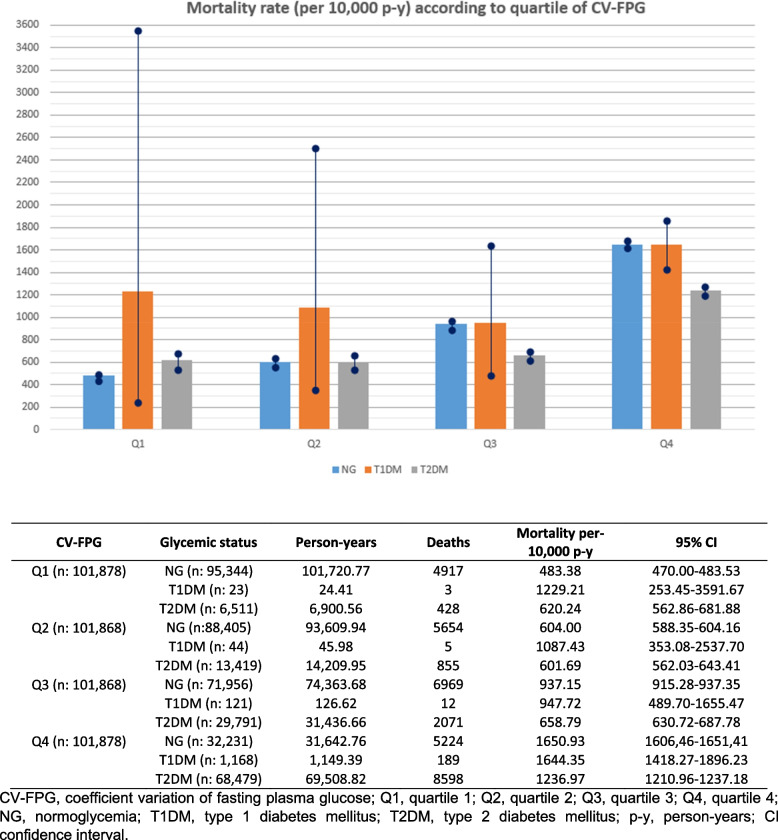
Mortality rates according to quartile of CV-FPG and glycemic status at baseline

During follow-up, 34,925 patients died (14,999 women and 19,926 men), with an all-cause mortality rate of 822.3 per 10,000 person-years (95% CI, 813.7 to 822.3) (739 per 10,000; 95% CI 728.7 to 739.0 in women and 967.1 per 10,000; 95% CI 951.7 to 967.2 in men). Mortality rates by glycemic status are shown in Fig. 2.

Compared with survivors, people who died were more likely to be male, older, ex-smokers, hypertensive, and had a more frequent history of Alzheimer’s disease, cancer, diabetes, chronic obstructive pulmonary disease (COPD), COVID-19 disease, cardiovascular disease (CVD), heart failure, atrial fibrillation, chronic kidney disease (CKD), and use of aspirin, anticoagulants, beta-blockers, and insulin. They also had lower mean body mass index (BMI), lower mean systolic and diastolic blood pressure, and higher mean baseline FPG (Table 2). The highest quartile of CV-FPG was significantly more common in the deceased group (40.1% vs. 23.6%; chi-square: 4,654.5; OR crude: 2.17 (CI 95%, 2,12 to 2,22); *p* < 0.001).

**Table 2:** Baseline sociodemographic and anthropometric measurements and clinical factors of all study participants and by survival status

Variables	Survivors (*N* = 372,567)	Deceased (*N* = 34,925)	*p* value
Age, mean (SD)	83.1 (5.4)	87.9 (6.1)	< 0.001
Sex male, n (%)	135,092 (36.3)	14,999 (42.9)	< 0.001
Barthel index, mean (SD)	80.5 (19.9)	68.2 (25.2)	< 0.001
***Grouped Barthel Index***			
Independent (100)	4/137,403 (0)	0/22,317 (0)	< 0.001
Slight dependence (61–99)	118,003/137,403 (85.9)	14,757/22,317 (66.1)	
Moderate dependence (41–60)	10,398/137,403 (7.6)	3,656/22,317 (16.4)	
Severe dependence (21–40)	4,257/137,403 (3.1)	2,086/22,317 (9.3)	
Complete dependence (< 20)	4,741/137,403 (3.5)	1,818/22,317 (8.1)	
Alcohol consumption, n (%)	4,233 (1.1)	522 (1.5)	< 0.001
Current smoking, n (%)	19,049 (5.1)	1,818 (5.2)	< 0.001
BMI, mean (SD)	28.7 (4.6)	28.1 (5)	< 0.001
Baseline SBP, mean (SD)	132.9 (16.1)	131.3 (17.2)	< 0.001
Baseline DBP, mean (SD)	74 (9.4)	72 (9.8)	< 0.001
T2DM patients, n (%)	105,992 (28.4)	11,888 (34)	< 0.001
T1DM patients, n (%)	1,403 (0.4)	273 (0.8)	
Normoglycemia patients, n (%)	265,172 (71.2)	22,764 (65.2)	
History of CVD, n (%)	52,280 (14)	8,293 (23.7)	< 0.001
Heart failure, n (%)	23,049 (6.2)	6,061 (17.4)	< 0.001
Atrial Fibrillation, n (%)	57,412 (15.4)	9,305 (26.6)	< 0.001
Hypertension, n (%)	275,381 (73.9)	26,227 (75.1)	< 0.001
Dyslipidemia, n (%)	226,255 (60.7)	18,110 (51.9)	< 0.001
Solid cancer, n (%)	16,785 (4.5)	3,450 (9.9)	< 0.001
Myeloma, n (%)	674 (0.2)	186 (0.5)	< 0.001
Leukemia, n (%)	1,246 (0.3)	294 (0.8)	< 0.001
Lymphoma, n (%)	1,937 (0.5)	309 (0.9)	< 0.001
Dementia (Alzheimer’s disease), n (%)	26,245 (7)	7,855 (22.5)	< 0.001
COVID-19, n (%)	10,828 (2.9)	3,863 (11.1)	< 0.001
COPD, n (%)	39,629 (10.6)	4,898 (14)	< 0.001
CKD, n (%)	109,793 (29.5)	14,763 (42.2)	< 0.001
Statin use, n (%)	208,308 (55.9)	13,592 (38.9)	< 0.001
ACEI or ARB use, n (%)	234,428 (62.9)	17,102 (49)	< 0.001
Antiplatelet drug use, n (%)	97,336 (26.1)	10,075 (28.8)	< 0.001
Anticoagulant use, n (%)	66,669 (17.9)	9,643 (27.6)	< 0.001
Beta-blocker use, n (%)	80,557 (21.6)	8,460 (24.2)	< 0.001
Calcium antagonist use, n (%)	91,759 (24.6)	7,028 (20.1)	< 0.001
Metformin use, n (%)	76,489 (20.5)	5,877 (16.8)	< 0.001
Insulin use, n (%)	22,361 (6)	3,360 (9.6)	< 0.001
Baseline FPG level, mean (SD)	104.4 (29.2)	105.7 (35.3)	< 0.001
CV-FPG, mean (SD)	10.1 (8.4)	14 (11.5)	< 0.001
Quartile 1 of CV-FPG, n (%)	96,530 (25.9)	5,348 (15.3)	< 0.001
Quartile 2 of CV-FPG, n (%)	95,353 (25.6)	6,514 (18.7)	
Quartile 3 of CV-FPG, n (%)	92,816 (24.9)	9,052 (25.9)	
Quartile 4 of CV-FPG, n (%)	87,861 (23.6)	14,010 (40.1)	

*CVD* cardiovascular disease (myocardial infarction, stroke, or peripheral artery disease), *CKD* chronic kidney disease (CKD-EPI < 60 ml/min/1.73 m^2^ and/or albumin/creatinine ratio ≥ 30 mg/g (≥ 3 mg/mmol)), *FPG* fasting plasma glucose, *CV-FPG* coefficient of variation of fasting plasma glucose, *BMI* body mass index, *SBP* systolic blood pressure, *DBP* diastolic blood pressure, *T2DM* type 2 diabetes mellitus, *T1DM* type 1 diabetes mellitus, *ACEI* angiotensin-converting enzyme inhibitor, *ARB* angiotensin II receptor blocker

The distribution of key clinical characteristics across quartiles of CV-FPG showed a higher burden of disease and vascular disease in the more extreme quartiles (3 and 4). Obviously, participants with T1DM and T2DM were more likely to be in quartile 4, and this circumstance could explain, at least in part, the high burden of vascular disease (Table 3). This fact justifies an adjustment for basal FPG in the multivariate models, as will be seen later.

**Table 3:** Baseline factors of 407,492 subjects grouped by quartiles of the coefficient of variation of FPG levels

**Variables**	**Quartiles of CV-FPG**				
	**1 (lowest)**	**2**	**3**	**4 (highest)**	***p* value**
Quartiles range	≤ 5,2635	5.2636–7.8577	7.8578–12.3739	≥ 12.3740	
N	101,878	101,868	101,868	101,878	
**Anthropometric and clinical variables**					
Male sex, n (%)	35,803 (35.1)	36,077 (35.4)	37,660 (37)	40,548 (39.8)	< 0.001^*^
Age, mean (SD)	82.9 (5.5)	83.2 (5.6)	83.7 (5.7)	84.1 (5.8)	< 0.001
Current smoking, n (%)	4,400 (4.3)	4,876 (4.8)	5,456 (5.4)	6,135 (6.0)	< 0.001^*^
Alcohol consumption, n (%)	929 (0.9)	1,065 (1)	1,252 (1.2)	1,509 (1.5)	< 0.001
BMI, mean (SD)	28.2 (4.4)	28.4 (4.5)	28.7 (4.7)	29.1 (4.9)	< 0.001
Baseline SBP, mean (SD)	132.4 (16.0)	132.5 (16.0)	132.7 (16.1)	133.3 (16.6)	< 0.001
Baseline DBP, mean (SD)	74.2 (9.3)	74.1 (9.4)	73.9 (9.4)	73.1 (9.6)	< 0.001
Hypertension, n (%)	70,539 (69.2)	73,477 (72.1)	76,989 (75.6)	80,596 (79.1)	< 0.001^*^
Dyslipidemia, n (%)	60,345 (59.2)	61,692 (60.6)	61,339 (60.2)	60,983 (59.9)	< 0.001
**Glycemic status**					
Normoglycemia, n (%)	95,344 (93.6)	88,404 (86.8)	71,956 (70.6)	32,227 (31.6)	< 0.001^*^
T2DM, n (%)	6,507 (6.4)	13,414 (13.2)	29,768 (29.2)	68,188 (66.9)	
T1DM, n (%)	27 (0.0)	49 (0.0)	144 (0.1)	1,456 (1.4)	
**Cardiovascular status**					
Previous CVD, n (%)	11,601 (11.4)	13,003 (12.8)	15,216 (14.9)	20,752 (20.4)	< 0.001^*^
Previous Heart failure, n (%)	4,349 (4.3)	5,523 (5.4)	7,543 (7.4)	11,695 (11.5)	< 0.001^*^
Atrial Fibrillation, n (%)	13,032 (12.8)	14,875 (14.6)	17,652 (17.3)	21,157 (20.8)	< 0.001^*^
CKD, n (%)	24,065 (23.6)	27,827 (26.8)	31,022 (30.5)	42,153 (41.4)	< 0.001^*^
**Other clinical conditions**					
Dementia (Alzheimer’s disease), n (%)	5,941 (5.8)	7,014 (6.9)	8,990 (8.8)	12,155 (11.9)	< 0.001^*^
COVID-19 during 2020, n (%)	2,926 (2.9)	3,176 (3.1)	3,853 (3.8)	4,736 (4.6)	< 0.001^*^
COPD, n (%)	9,407 (9.2)	10,508 (10.3)	11,835 (11.6)	12,777 (12.5)	< 0.001^*^
Solid Cancer, n (%)	4,369 (4.3)	4,615 (4.5)	5,227 (5.2)	5,984 (5.9)	< 0.001^*^
**Medication profile**					
Statin, n (%)	50,988 (50.0)	53,737 (52.8)	55,884 (54.9)	61,285 (60.2)	< 0.001^*^
ACEI or ARB, n (%)	59,143 (58.1)	61,404 (60.3)	63,555 (62.4)	67,422 (66.2)	< 0.001^*^
Antiplatelet drug, n (%)	21,920 (21.5)	24,049 (23.6)	26,801 (26.3)	34,639 (34.0)	< 0.001^*^
Anticoagulant, n (%)	15,304 (15.0)	17,120 (16.8)	20,038 (19.7)	23,847 (23.4)	< 0.001^*^
Diuretics, n (%)	41,458 (40.7)	45,051 (44.2)	48,956 (48.1)	53,874 (52.9)	< 0.001^*^
Loop diuretics, n (%)	12,118 (11.9)	14,786 (14.5)	18,689 (18.3)	26,022 (25.5)	< 0.001^*^
Beta-blocker use, n (%)	18,474 (18.1)	20,467 (20.1)	23,084 (22.7)	26,989 (26.5)	< 0.001^*^
Calcium antagonist, n (%)	20,720 (20.3)	23,024 (22.6)	25,218 (24.8)	29,822 (29.3)	< 0.001^*^
Metformin, n (%)	4,646 (4.6)	9,792 (9.6)	22,014 (21.6)	45,911 (45.1)	< 0.001^*^
DPP4-Inhibitor	1,219 (1.2)	3,064 (3.0)	8,986 (8.8)	31,792 (31.2)	< 0.001^*^
SGLT2-Is	171 (0.2)	441 (0.4)	1,332 (1.3)	5,371 (5.3)	< 0.001^*^
Insulin use, n (%)	228 (0.0)	500 (0.5)	1,762 (1.7)	23,230 (22.8)	< 0.001^*^

*FPG-CV* coefficient of variation of fasting plasma glucose, *BMI* body mass index, *SBP* systolic blood pressure, *DBP* diastolic blood pressure, *T2DM* type 2 diabetes mellitus, *T1DM* type 1 diabetes mellitus, *CVD* cardiovascular disease (myocardial infarction, stroke, and peripheral artery disease), *CKD* chronic kidney disease, *COVID-19* coronavirus disease 2019, *COPD* chronic obstructive pulmonary disease, *ACEI* angiotensin-converting enzyme inhibitor, *ARB* angiotensin II receptor blocker, *DPP4-inhibitor* dipeptidyl peptidase-4 inhibitor, *SGLT2-Is* sodium-glucose cotransporter-2 inhibitor

^*^p value for linear trend across quartiles of CV-FPG

The adjusted effect of FPG variability on all-cause mortality was examined with four models, as shown in Table 4. Compared with the first quartile of CV-FPG, the second, third and fourth quartiles showed a statistically significant increase in all-cause mortality in models 1, 2 and 3. In the full model (Model 4), given the interaction between quartiles of CV-FPG and glycemic status, the results are presented according to three glycemic levels: normoglycemia, T1DM, and T2DM. In normoglycemic subjects, the second, third and fourth quartiles achieved a statistically significant increase in all-cause mortality compared with the first quartile. In contrast, in people with T1DM, there was no significant increase in any quartile. In people with T2DM, only the highest quartile showed a statistically significant increase compared with the first. In quantitative terms, the highest mortality risk was for the fourth quartile in subjects with normoglycemia (OR, 2.30; 95% CI, 2.20 to 2.41), followed by patients with T2DM (OR, 1.88; 95% CI, 1.69 to 2.09).

**Table 4:** The odds ratios (ORs) of all-cause mortality grouped by quartiles of the coefficient of variation of FPG among 407,492 subjects with at least three FPG measurements

	**Quartiles of CV of FPG**			
	**1 (lowest)**	**2**	**3**	**4 (highest)**
N	101,878	101,868	101,868	101,878
Deaths for All causes, n (%)	5,348 (5.2)	6,514 (6.4)	9,052 (8.9)	14,011 (13,8)
**Model 1**	1	1.19 (1.14–1.24)^*^	1.60 (1.54–1.66)^*^	2.54 (2.45–2.62)^*^
**Model 2**	1	1.17 (1.12–1.21)^*^	1.52 (1.46–1.58)^*^	2.29 (2.21–2.37)^*^
**Model 3**	1	1.16 (1.11–1.20)^*^	1.47 (1.42–1.53)^*^	2.17 (2.09–2.26)^*^
**Model 4**				
NG	1	1.17 (1.12–1.21)^*^	1.57 (1.50–1.63)^*^	2.30 (2.20–2.41)^*^
T1DM	1	1.69 (0.32–8.94)	1.57 (0.36–6.94)	2.81 (0.73–10.85)
T2DM	1	1.02 (0.90–1.16)	1.11 (0.99–1.24)	1.88 (1.69–2.09)^*^

Model 1: adjusted for age and sex. Model 2: adjusted for age, sex, history of cardiovascular disease, heart failure, COVID-19 infection, and solid cancer. Model 3: adjusted for variables in model 2 plus glycemic status, hypertension, atrial fibrillation, COPD, CKD, use of tobacco, dyslipidemia, antiplatelet use, statin use, and SRA use. Model 4: Model 3 plus dementia (Alzheimer’s disease), and basal value of FPG stratified by glycemic status

*N* number of persons included in the analysis for each group, *FPG* fasting plasma glucose, *CV* coefficient of variation, *NG* normoglycemia

^*^p < 0.001

Three secondary sensitivity analysis of the logistic regression were carried out (Tables 5, 6 and 7). The first of them which excluded persons with a history of cancer is shown in Table 5. The ORs overlap with the primary analysis, with a similar magnitude of association in each quartile and model. The absence of interaction between quartiles of CV-FPG and glycemic status did not allow the comparison of ORs between normoglycemia, T1DM and T2DM. The second sensitivity analysis (Table 6), which excluded patients with a history of cancer and those who died from COVID-19, showed that the results were consistent, as small changes in the OR were observed. However, when the results were stratified by glycemic status, T1DM had no significant effect on mortality, as in the primary analysis, and T2DM only in the third and fourth quartiles of CV-FPG.

**Table 5:** The odds ratios (ORs) of all-cause mortality grouped by quartiles of the coefficient of variation of FPG excluding patients with a history of cancer

	**Quartiles of CV of FPG**			
	**1 (lowest)**	**2**	**3**	**4 (highest)**
N	82,638	81,720	80,440	79,425
Deaths for All causes, n (%)	3,887 (4.7)	4,760 (5.8)	6,572 (8.2)	10,198 (12,8)
**Model 1**	1	1.21 (1.15–1.26)^*^	1.62 (1.55–1.69)^*^	2.59 (2.49–2.69)^*^
**Model 2**	1	1.19 (1.13–1.24)^*^	1.55 (1.48–1.61)^*^	2.35 (2.25–2.44)^*^
**Model 3**	1	1.19 (1.13–1.24)^*^	1.55 (1.48–1.62)^*^	2.38 (2.27–2.49)^*^
**Model 4**	1	1.17 (1.12–1.22)^*^	1.52 (1.46–1.58)^*^	2.34 (2.24–2.43)^*^

Model 1: adjusted for age and sex. Model 2: adjusted for age, sex, history of cardiovascular disease, heart failure, and COVID-19 infection. Model 3: adjusted for variables in model 2 plus glycemic status, hypertension, atrial fibrillation, COPD, CKD, use of tobacco, dyslipidemia, antiplatelet use, statin use, and SRA use. Model 4: interaction between quartiles of CV-FPG and glycemic status was negative (*p* = 0.261); include model 3 plus dementia (Alzheimer`s disease) plus basal value of FPG

*N* number of persons included in the analysis for each group, *FPG* fasting plasma glucose, *CV* coefficient of variation

^*^p < 0.001

**Table 6:** The odds ratios (ORs) of all-cause mortality grouped by quartiles of the coefficient of variation of FPG excluding patients with a history of cancer and those who died from COVID-19

	**Quartiles of CV of FPG**			
	**1 (lowest)**	**2**	**3**	**4 (highest)**
N	81,750	80,442	78,890	77,190
Deaths for All causes, n (%)	2,897 (3.6)	3,582 (4.5)	5,149 (6.5)	8,139 (10,5)
**Model 1**	1	1.22 (1.15–1.28)^*^	1.70 (1.62–1.78)^*^	2.76 (2.64–2.89)^*^
**Model 2**	1	1.20 (1.14–1.26)^*^	1.64 (1.56–1.72)^*^	2.55 (2.44–2.67)^*^
**Model 3**	1	1.20 (1.14–1.26)^*^	1.65 (1.57–1.74)^*^	2.63 (2.50–2.77)^*^
**Model 4**				
**NG**	1	1.20 (1.14–1.26)^*^	1.70 (1.63–1.79)^*^	2.61 (2.48–2.76)^*^
**T1DM**	1	1.97 (0.28–13.73)	1.53 (0.25–9.19)	2.43 (0.46–12.92)
**T2DM**	1	0.98 (0.84–1.15)	1.15 (1.01–1.32)^*^	2.06 (1.81–2.34)^*^

Model 1: adjusted for age and sex. Model 2: adjusted for age, sex, history of cardiovascular disease, heart failure, and COVID-19 infection. Model 3: adjusted for variables in model 2 plus glycemic status, hypertension, atrial fibrillation, COPD, CKD, use of tobacco, dyslipidemia, antiplatelet use, statin use, and SRA use. Model 4: Model 3 plus dementia (Alzheimer’s disease), basal value of FPG, stratified by glycemic status

*N* number of persons included in the analysis for each group, *FPG* fasting plasma glucose, *CV* coefficient of variation

^*^p < 0.001

**Table 7:** Odds ratios (ORs) for all-cause mortality grouped by quartiles of the coefficient of variation of FPG in patients with COVID-19 infection and without a history of cancer

	**Quartiles of CV of FPG**			
	**1 (lowest)**	**2**	**3**	**4 (highest)**
N	2,325	2,512	2,987	3,646
Deaths for All causes, n (%)	544 (23.4)	562 (22.4)	737 (24.7)	1,065 (29,2)
**Model 1**	1	0.94 (0.82–1.08)	1.02 (0.89–1.16)	1.28 (1.13–1.45)^*^
**Model 2**	1	0.94 (0.82–1.07)	1.01 (0.88–1.14)	1.23 (1.09–1.39)^*^
**Model 3**	1	0.92 (0.80–1.05)	0.95 (0.83–1.09)	1.08 (0.94–1.24)
**Model 4**	1	0.84 (0.74–0.96)	0.91 (0.80–1.04)	0.96 (0.82–1.11)

Model 1: adjusted for age and sex. Model 2: adjusted for age, sex, history of cardiovascular disease, and heart failure. Model 3: adjusted for variables in model 2 plus glycemic status, hypertension, atrial fibrillation, COPD, CKD, use of tobacco, dyslipidemia, antiplatelet use, statin use, and SRA use. Model 4: No interaction between quartiles CV-FPG and glycemic status (*p* = 0.192); model 3 plus dementia (Alzheimer’s disease) and basal value of FPG

*N* number of persons included in the analysis for each group, *FPG* fasting plasma glucose, *CV* coefficient of variation

^*^p < 0.001

Finally, we analysed the effect of GV on all-cause mortality among participants infected with SARS-CoV-2 to determine whether there were differences with respect to the global population analysis. As shown in Table 7, the more adjusted models (3 and 4) did not show significant associations between GV and mortality risk. In this case, the absence of interaction between quartiles of CV-FPG and glycemic status also did not allow to compare ORs between stratified glycaemic status.

## Discussion

It is well established that long-term GV is an independent predictor of all-cause mortality in patients with DM [23]. However, there is still insufficient evidence in the population without DM, at least when using quartiles of CV-FPG [14]. In this regard, a prospective cohort analysis in the Antihypertensive and Lipid-Lowering Treatment to Prevent Heart Attack Trial (ALLHAT) showed a similar effect of GV in those participants without DM and higher CV-FPG when used as a continuous variable, with a hazard ratio for all-cause mortality of 1.032 (95% CI, 1.014 to 1.049) in the most fully adjusted model [24].

Early analyses by our group showed that in individuals with prediabetes or T2DM, the fourth quartile of CV-FPG had a significant association with all-cause mortality after simple and full adjustment [16].

To our knowledge, only one study has examined the association between GV and mortality in the elderly population. This was a retrospective cohort study using the Health Improvement Network (THIN) database, which included 54,803 individuals aged 70 years and older in 587 UK primary care practices. All were diagnosed with diabetes, and GV was assessed by HbA1c variability over time. Higher HbA1c variability was associated with mortality, and a J-shaped curve was observed in the relationship between HbA1c thresholds and mortality [25].

Our study was conducted in the entire population aged 75 years and over 29.3% of them were diagnosed with DM at baseline. Regarding the type of DM, the crude mortality rates showed high figures for T1DM compared with T2DM, probably because of the most frequent use of insulin, which is most associated with glycemic variability, and because T2DM patients are more likely (Table 3) to take medications such as DPP-4 inhibitors and ISGLT-2, which have been shown to reduce vascular complications (e.g., heart failure and CKD). When the primary and secondary multivariate sensitivity analyses were carried out, patients with T1DM had a high propensity for all-cause mortality for the most extreme quartiles of CV-FPG. However, this did not reach statistical significance due to the small sample size (0.4% of the study population). Patients with T2DM showed an increased mortality risk for the highest quartile in the primary and secondary sensitivity analyses. In the latter case, the increase in mortality was also significantly associated with the third quartile, but the magnitude was small. The patients with normoglycemia showed lower crude mortality rates than those with other glycemic status. However, the multivariate primary analysis showed an increased mortality risk for any quartile of CV-FPG. The same phenomenon was observed in the secondary sensitivity analysis but with even larger magnitudes of association. These findings also highlight the importance of glycemic variability, including small CV-FPG, in both normoglycemic and T2DM participants, as we had previously found.

Alzheimer’s disease is often associated with causes of mortality [26] so we considered including this disease in the fully adjusted model. An analysis excluding patients with Alzheimer’s disease (8.4% of the sample population) could be a valid option. However, this would lead to the loss of an important variable that acts as a confounder between GV (exposure) and mortality (event). Although Alzheimer’s disease is associated with both GV [27–29] and mortality [26, 30], it is not a part of the causal pathway. These three aspects are necessary for a variable to be considered a confounder [31].

With respect to physiopathology, higher GV has been associated with high protein expression of markers such as Wnt1 [32]. The Wnt signaling pathway causes at least two factors associated with mortality: first, it favours vascular calcification and regulates key aspects of vascular disease [33], as this calcification is more prevalent in the elderly than in the young and is highly associated with cardiovascular disease mortality [34]; second, the Wnt signaling pathway causes susceptibility to cancer [35]. On the other hand, elderly people tend to develop mitochondrial dysfunction, which increases oxidative damage during aging and metabolic diseases [36]. Additionally, GV “per se” has been associated with oxidative stress in patients with T2DM and hypertension [37], which increases the inflammatory response, vascular calcification [38], and endothelial damage, all of which lead to vascular complications and mortality. In addition, the restrictions to maintain a dietary regimen due to lockdown in the older people may explain a possible modification of the sequence of macronutrients and vegetables intake. This situation has been highlighted as inductor of GV given the modifying the time to glucose elevation, the glucose curve magnitude, and the glucose decay time [39].

Data from patients with acute injury, such as intracerebral hemorrhage, analysed by a recent meta-analysis [40], showed that those who had a higher category of standard deviation of blood glucose were associated with a higher risk of mortality (RR: 2.39, 95% CI: 1.79 to 3.19, *p* < 0.001). Other injuries, such as SARS-CoV-2 infection with acute respiratory distress syndrome, have shown similar results in a recent study of intensive care unit (ICU) patients: CV-FPG measured daily showed an adjusted OR for mortality of 12.83 (95% CI, 1.24–132.58) [41]. In our study, in patients with SARS-CoV-2 infection, GV was associated with a lower effect on all-cause mortality. This finding could be because in our case, we included a broad spectrum of patients with COVID-19: nonhospitalized, admitted to the ICU and hospitalized in beds outside the ICU. Measures to contain the COVID-19 outbreak reduced outdoor physical activity among older people [42, 43]. There was also a significant decrease in social participation among older people [44]. In addition, health and social support services were reduced with a downward trend in attendance at medical appointments [45, 46]. These conditions may explain the increase in GV and its association with mortality.

Our study has several strengths, including its robust design to minimize bias and the large number of patients with diabetes, diabetes plus hypertension, and normoglycemia. In addition, to our knowledge, our study is the first to examine the relationship between variability in FPG and all-cause mortality in elderly patients with differences in glycemic status in southern European countries. This aspect is especially relevant, given the possible lower effect of GV on all-cause mortality in countries with healthier lifestyles [47] and better glycemic control than other countries participating in the EUROASPIRE IV survey [48].

This study has some limitations. First, we included patients with differences in glycemic status, and the analyses could not be adjusted for variables such as mean HbA1c, duration of diabetes, diabetic nephropathy, diabetes treatments, or microalbuminuria, as in other studies. Second, the number of patients with T1DM was small and this situation reduced the power to find statistical associations with mortality in the multivariate analysis. Third, we did not have information on the cause of death, which would have enabled us to verify that mortality is primarily accounted for by cardiovascular disease, given the known association between GV and macrovascular complications. Fourth, we did not record hypoglycemia episodes and could not assess their association with mortality. Fifth, we could not study GV measured with CV-HbA1c, given that few persons with normoglycemia or IGT had at least three HbA1c measurements during follow-up. Sixth, given the observational nature of the present study, individuals with higher GV and lower GV were dissimilar. Therefore, adjusting for differences in both groups in the multivariate analysis was necessary to obtain an accurate picture of the association between all-cause mortality and GV. Propensity score matching (PSM) would be an appropriate alternative that would yield less biased results than standard methods such as logistic regression. However, given that propensity scores can only control for observed confounders, they cannot be counted upon to balance unobserved covariates.

## Conclusion

GV has clear implications for clinical practice during the first year of the COVID-19 pandemic, as its assessment as a risk prediction tool should be included in the routine follow-up of the elderly and in a comprehensive geriatric assessment. Electronic medical records can incorporate tools that allow its calculation, and with this information, clinicians will have a broader view of the medium- and long-term prognosis of their patients**.**

### Supplementary Information

Supplementary Material 1.

## Data Availability

The datasets used or analysed during the current study are available from the corresponding author upon reasonable request.

## Abbreviations

CKD: Chronic kidney disease
COPD: Chronic obstructive pulmonary disease
COVID-19: Coronavirus disease 2019
EHRs: Electronic health records
GV: Glucose variability
T2DM: Type 2 diabetes mellitus
T1DM: Type 1 diabetes mellitus
ICPC-2: The International Classification of Primary Care (ICPC-2).
FPG: Fasting plasma glucose
INDEF: Office of National Statistics death registrations
CV: Coefficient of variation
CV-FPG: Coefficient of variation of fasting plasma glucose
