# Supplementary figures and images for: Effect of glucose variability on the mortality of adults aged 75 years and over during the first year of the COVID-19 pandemic
Source: BMC Geriatr. 2024 Jun 20;24:533. doi: 10.1186/s12877-024-05149-0 (PMC11188234; doi:10.1186/s12877-024-05149-0)

**SUPPLEMENTARY INFORMATION**


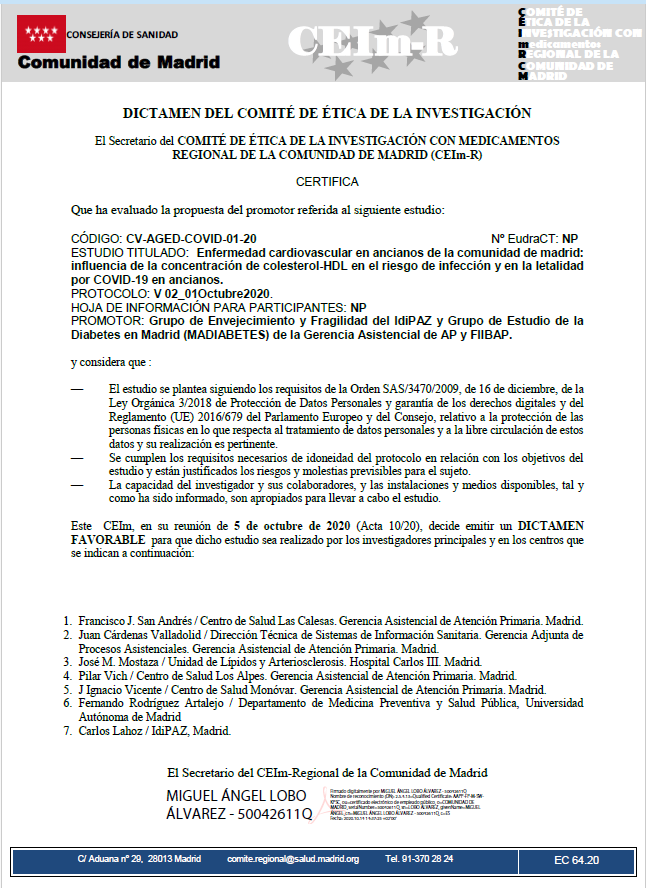

Supplement: Supplementary file 1 — Supplementary Material 1. [file 12877_2024_5149_MOESM1_ESM.docx]
